# Multi-shell diffusion MRI of the fornix as a biomarker for cognition in Alzheimer’s disease

**DOI:** 10.1016/j.mri.2024.03.030

**Published:** 2024-03-21

**Authors:** Ken Sakaie, Katherine Koenig, Alan Lerner, Brian Appleby, Paula Ogrocki, Jagan A. Pillai, Stephen Rao, James B. Leverenz, Mark J. Lowe

**Affiliations:** a Imaging Institute, The Cleveland Clinic, 9500 Euclid Ave, Mail code U-15, Cleveland, OH 44195, USA; b Department of Neurology, Case Western Reserve University School of Medicine, Cleveland, OH, USA; c Lou Ruvo Center for Brain Health, The Cleveland Clinic, 9500 Euclid Ave, Mail code U-10, Cleveland, OH 44195, USA

**Keywords:** Diffusion MRI, Fornix, Partial volume effects, Mild cognitive impairment, Alzheimer’s disease, Episodic memory

## Abstract

**Background and purpose:**

A substantial fraction of those who had Alzheimer’s Disease (AD) pathology on autopsy did not have dementia in life. While biomarkers for AD pathology are well-developed, biomarkers specific to cognitive domains affected by early AD are lagging. Diffusion MRI (dMRI) of the fornix is a candidate biomarker for early AD-related cognitive changes but is susceptible to bias due to partial volume averaging (PVA) with cerebrospinal fluid. The purpose of this work is to leverage multi-shell dMRI to correct for PVA and to evaluate PVA-corrected dMRI measures in fornix as a biomarker for cognition in AD.

**Methods:**

Thirty-three participants in the Cleveland Alzheimer’s Disease Research Center (CADRC) (19 with normal cognition (NC), 10 with mild cognitive impairment (MCI), 4 with dementia due to AD) were enrolled in this study. Multi-shell dMRI was acquired, and voxelwise fits were performed with two models: 1) diffusion tensor imaging (DTI) that was corrected for PVA and 2) neurite orientation dispersion and density imaging (NODDI). Values of tissue integrity in fornix were correlated with neuropsychological scores taken from the Uniform Data Set (UDS), including the UDS Global Composite 5 score (UDSGC5).

**Results:**

Statistically significant correlations were found between the UDSGC5 and PVA-corrected measure of mean diffusivity (MDc, *r* = −0.35, *p* < 0.05) from DTI and the intracelluar volume fraction (ficvf, *r* = 0.37, *p* < 0.04) from NODDI. A sensitivity analysis showed that the relationship to MDc was driven by episodic memory, which is often affected early in AD, and language.

**Conclusion:**

This cross-sectional study suggests that multi-shell dMRI of the fornix that has been corrected for PVA is a potential biomarker for early cognitive domain changes in AD. A longitudinal study will be necessary to determine if the imaging measure can predict cognitive decline.

## 1. Introduction

The neuropathologic hallmarks of Alzheimer’s disease (AD) are accumulation of amyloid beta (Aβ) and tau neurotangles and neurites, but the presence of such neuropathology does not necessarily result in dementia. For example, a recent meta-analysis has found that a third of subjects with AD neuropathologic changes at autopsy did not have clinical dementia [1]. Biomarkers that can improve the ability to identify those at risk of cognitive decline are needed, as they have the potential to help patients and their families understand the patient’s medical condition, allow patients and physicians to make informed treatment decisions and contribute to the success of clinical trials. Although biomarkers within the amyloid-tau-neurodegeneration (ATN) framework [2] constitute substantial progress towards measuring neuropathologic changes associated with AD, these biomarkers improve prediction of cognitive decline by only a small amount in individuals without dementia [3].

The structure and function of the fornix suggest that early damage, captured by diffusion MRI (dMRI), may serve as a predictive biomarker for early cognitive decline in AD. The fornix is the primary efferent of the hippocampus and plays an important role in episodic memory [4], a cognitive domain that exhibits deficits early in AD [5]. The critical role of the fornix is reflected by its potential use as a therapeutic target to reverse AD-related cognitive decline [6]. Although the hippocampus exhibits AD-specific pathology at early stages of the disease [7], hippocampal volume is the last of the ATN biomarkers to become abnormal [8]. Atrophy of the fornix is related to cognitive decline [9,10]. However, atrophy reflects neuronal loss, the end stage of neurodegeneration, and so may have limited utility for indicating early-stage neuronal dysfunction. dMRI provides measures of tissue microstructure that can reflect subtle injury presumably before irreversible neuronal loss. dMRI changes in the fornix are of particular interest because injury can manifest early, even among pre-symptomatic carriers of familial AD mutations and in pre-clinical AD [11,12]. dMRI of fornix predicts overall cognition [10] and episodic memory [9], suggesting that dMRI of this region is a candidate marker for cognitive decline in early AD.

The sensitivity of the fornix may be due to the fact that the body of the fornix does not overlap other eloquent regions when imaged at spatial resolution that is readily achievable by dMRI [13]. For example, the earliest Braak stage entails the formation of neurofibrillary tangles in layer 2 of the entorhinal cortex [7], with the appearance of tangles in the hippocampus at a later stage [14]. From the standpoint of pathology, this finding suggests the perforant pathway as a reasonable candidate for study by in vivo imaging, as it connects layer 2 of the entorhinal cortex and the hippocampus. However, differentiating measures of the perforant pathway from adjacent pathways, such as the angular bundle, requires ultra-high spatial resolution that is challenging to perform in vivo in humans [15].

Partial volume averaging (PVA) between fornix and cerebrospinal fluid (CSF) must be accounted for when performing dMRI measurements of the fornix. Mean thickness at the crus of the fornix is 2.7 mm [16]. As typical spatial resolution for dMRI is 2.0 mm, most voxels in the fornix will contain a mixture of tissue and CSF. Although CSF is not eloquent tissue, diffusivity in tissue and CSF differ substantially. The resulting bias in dMRI measurements can lead to misleading inferences in populations with mild cognitive impairment (MCI) [17]. Corrections for PVA are best achieved by voxelwise modelling of the dMRI signal [18]. Previous work investigating dMRI in the fornix relied on dMRI acquisitions with a single nonzero diffusion weighting together with modelling that uses spatial regularization to handle degeneracies when fitting the data [19]. Subsequent work has shown that dMRI acquisitions that acquire multiple nonzero diffusion weightings, so-called multishell dMRI, enable more reliable modelling [20].

To our knowledge, there is no work investigating PVA corrections of multishell dMRI in fornix and their correlations with cognitive measures in an older sample ranging from normal cognition to MCI to AD. In this study, we investigate relationships between neuropsychological test performance and multishell dMRI that has been corrected for PVA. Two PVA modelling approaches are examined. One uses a diffusion tensor to represent tissue and an isotropic component to represent CSF [21]. The other is the neurite orientation dispersion and density imaging (NODDI) model [22]. Each of these models generates several measures of tissue integrity, and we sought to determine which shows the strongest relationship to cognitive measures.

## 2. Material and methods

### 2.1. Sample

Participants were selected from enrollees in the Cleveland Alzheimer’s Disease Research Center (CADRC). Selection was based on diagnosis (normal cognition (NC), MCI due to AD or dementia due to AD) and on the availability of MRI images. Diagnosis was based on the CADRC consensus panel of neurologists, geriatric psychiatrists and neuropsychologists reviewing clinical history as well as performance on the UDS cognitive test battery [23]. All participants were enrolled into the IRB-approved CADRC protocol after giving written informed consent.

### 2.2. Neuropsychological battery

As part of the CADRC, participants completed the neuropsychological battery specified in the Uniform Data Set (UDS) version 3 [23]. Tests and associated variables were Craft Story (immediate and delayed recall), Benson Complex Figure Test (copy and delayed recall), category fluency (animals and vegetables), Multilingual Naming Test (total score), phonetic fluency (total correct for letters F and L), Trail Making Test (parts A and B) and Number Span (total forward and total backward). Test scores were converted to z-scores using the UDS3 norms calculator [23], which accounts for effects due to gender, age and education. Using the procedure described by Dodge et al. [24], z-scores were used to calculate the UDS Global Composite-5 score (UDSGC5) and domain-specific composite scores of memory, language, attention, executive function and visuospatial performance.

### 2.3. Image acquisition

Each subject underwent an MRI exam that occurred 29 ± 38 days (mean ± standard deviation) after cognitive testing. All imaging was performed on a Siemens Prisma 3 T MRI with a standard 32 channel head coil (Siemens Healthineers, Erlangen, Germany). Imaging included anatomical and diffusion scans based on the advanced ADNI3 advanced protocol (https://adni.loni.usc.edu/). The anatomical scan was an MPRAGE (sagittal, 1 mm isotropic voxels, whole-brain coverage). The diffusion scan (axial, 116 × 116 × 78 matrix, 2 mm isotropic voxels, TE/ TR = 71/3300 msec, partial fourier factor 6/8, slice acceleration factor 3, posterior to anterior phase encoding direction, TA = 7 min 12 s) was a multishell acquisition (13/6/48/60 b = 0/500/1000/2000 s/mm^2^). A single reverse-phase encoded image volume without diffusion weighting was acquired to correct for susceptibility distortion using topup from FSL [25]. Diffusion images were corrected for motion and eddy current distortion using eddy [26] from FSL [25].

### 2.4. Image analysis

The fornix was segmented from the MPRAGE using a deep-learning based tool [27] from freesurfer [28] and coregistered to dMRI space using align-anat-*epi* [29] from AFNI [30]. dMRI data was fit on a voxelwise basis to three models: 1) a standard diffusion tensor, uncorrected for PVA (DTIu), using the dtifit tool from FSL with the weighted least squares option [25], 2) a PVA-corrected diffusion tensor (DTIc), using an algorithm developed Hoy et al. [31] and implemented by Henriques et al. [32] with dipy [33] and 3) the NODDI model [22] implemented with the NODDI toolbox (http://mig.cs.ucl.ac.uk). From the DTIu model, four maps of tissue integrity were generated—fractional anisotropy (FAu), mean diffusivity (MDu), longitudinal diffusivity (LDu) and transverse diffusivity (TDu). The DTIc model results in corresponding maps that have been corrected for partial volume averaging (PVA) with CSF (FAc, MDc, LDc, TDc) and the signal fraction attributed to CSF (fCSF), a measure of PVA. For the DTIu and DTIc models, only signal with b < 1500 s/mm^2^ was used in order to limit bias from nongaussian decay [34]. The NODDI model generates maps of two parameters that represent the density and dispersion of axons: intracellular volume fraction (ficvf) and orientation dispersion index (odi). The NODDI model also generates a measure of PVA with CSF, in which isotropic diffusion is expected (fiso). In all, each voxel has associated with it 12 dMRI parameters. The median across voxels within the fornix was taken for parameters from the DTIu model and for the PVA estimates (fCSF and fiso). For tissue microstructure measures from the DTIc model (FAc, MDc, LDc, TDc) and for the NODDI model (ficvf and odi), a weighted mean with the tissue fraction was taken [35]. The tissue fraction was defined as (1 – fCSF) for the DTIu model and as (1 – fiso) for the NODDI model.

### 2.5. Statistical analysis

One-way ANOVA with diagnosis as a categorical factor was used to compare neuropsychological test scores. As age was accounted for in the construction of the neuropsychological test scores, age was not included as a covariate. Post hoc comparisons used the Tukey-Kramer test. For dMRI measures, two-way ANOVA with diagnosis as a categorical factor and age as a continuous factor was used to account for age dependence in dMRI measures of fornix [36]. Partial correlation, with age as a covariate, was used to compare the UDSGC5 score to each of the 12 dMRI parameters. Tests with *p* < 0.05 were considered statistically significant. As this was an exploratory analysis, no corrections for multiple comparisons were performed. A sensitivity analysis was performed to determine if relationships were driven by performance in specific domains. Only dMRI parameters that showed a significant relationship to the UDSGC5 were considered in the sensitivity analysis. Partial correlation was calculated, with age as a covariate, between dMRI parameters and domain-specific composite scores for the sensitivity analysis.

## 3. Results

Thirty-three participants were included in the analysis: 19 NC, 10 MCI and 4 CE. Table 1 reports demographics, neuropsychological test scores and ANOVA results. A chi-squared test of proportions was used to compare proportions of females among groups. The NC group was significantly younger than both the MCI and AD groups (*p* < 0.011). The NC group scored higher on both the UDSGC5 and the memory composite score compared to MCI and AD (*p* < 0.001). The language composite score showed significant differences for all pairwise comparisons (p < 0.001). ANOVA tests of all imaging measures had *p* > 0.12.

Scatter plots of the UDSGC5 and each of the 12 dMRI parameters are shown in Fig. 1. Correlations with MDc (*r* = −0.35, *p* < 0.05) and with ficvf (*r* = 0.37, *p* < 0.04). Healthier tissue is associated with lower values of MD and with higher values of ficvf consistent with the direction of the relationships.

Table 2 shows results of the sensitivity analysis. The relationship between UDSGC5 and MDc is driven by memory (*r* = −0.53, *p* < 0.003) and by language (*r* = −0.47, *p* < 0.007). Correlations between specific domains and ficvf were not significant (*p* > 0.05).

## 4. Discussion

This study assessed the potential of dMRI measures of tissue integrity as biomarkers for cognition in AD. The primary finding was that MDc and ficvf correlate with the UDSGC5. White matter abnormalities have been found in preclinical AD subjects [37]. dMRI of injury to white matter pathways in general might prove useful in the context of AD [38]. Our findings align with previous studies that have shown that dMRI of fornix is particularly promising in the context of AD [39].

Association between performance in specific cognitive domains and dMRI has been noted previously in the context of AD. Associations between memory and dMRI measures have been found in a diverse array of brain regions. Lancaster et al. [40] found that LDu and MDu in medial temporal lobe could predict decline of episodic memory in a cohort of cognitively intact older adults that was enriched with subjects at high risk of AD. Nir et al. [41] found correlations between memory and diffusivity measures (LDu, TDu and RDu) in the cingulum, inferior fronto-occipital fasciculus and sagittal stratum, uncinate and external capsule, but correlations were not found between memory and FAu. Mayo et al. [42] found widespread correlations between memory and FAu, MDu and RDu in a large number of regions, but these correlations were only significant if both healthy adults and those with AD were considered together, not separately. Associations between language deficits and language performance have been observed in corpus callosum [43] and superior longitudinal fasciculus [44]. Elevated MD has been associated with neurofibrillary tangles but not Aβ plaques in a comparison of antemortem DTI and pathology [45].

A major trend in AD research is to identify biomarkers that can predict cognitive decline. A number of clinical trials are testing the efficacy of therapies in Aβ-positive subjects with normal cognition, but the variability of rates of cognitive decline put the success of these trials at risk [46]. Identifying biomarkers that can enrich trial populations with those at high risk of cognitive decline is therefore a high priority. Of particular interest are biomarkers that can predict risk prior to onset of cognitive decline. By construction, neuropsychological tests detect cognitive decline only after onset. While plasma biomarkers have been proposed as a cost-effective biomarker [46], it may prove important to develop alternative approaches. In this study, dMRI of the fornix was unable to differentiate NC, MCI and AD groups. However, it was able to detect more trends in cognition, reflected by the UDSGC5. Correlation between MDc and domains of memory and language might indicate sensitivity to early cognitive decline, as deficits in memory and language can occur early in AD [5,47]. Future work will pursue our long term goal to determine the utility of dMRI of fornix to predict cognitive decline prior to symptom onset.

One of the contributions of this work is its exploitation of recent advances in corrections for PVA. Previous work has shown that PVA must be accounted for on a voxelwise basis [18]. Without such corrections, group differences between subjects with NC and MCI are exaggerated [17]. However, this previous work [17,18] used a different PVA correction algorithm. The previous work used a spatial regularization approach [19] and a single-shell acquisition. More recent work has shown that signal modelling based on a multi-shell acquisition [31] is more reliable [20], and the multi-shell approach is used in this study. Two studies that use multishell acquisitions in combination with PVA correction have found correlations between memory and dMRI measures. Reas et al. [48] used restriction spectrum imaging, an alternative approach to NODDI, and found correlations between memory scores and neurite density measures in arcuate fasciculus. Parker et al. [49] demonstrated that NODDI correlated with memory performance more strongly than DTI. Although implementation of multi-shell acquisitions adds complexity to dMRI scans, the current ADNI3 protocol supports such an acquisition to facilitate widespread, harmonized use of such imaging.

dMRI measures may be useful for distinguishing different types of dementia. For example, dMRI measures in the nigroputaminal pathway may distinguish dementia with lewy bodies (DLB) from AD [50]. Hippocampal disorders that cause amnestic dementia, such as limbic age-associated TDP-43 encephalopathy (LATE), have been found to incur injury in the fornix in an animal model [51]. Although dMRI lacks specificity to pathology, it is possible that different pathways will be affected by different subtypes of dementia.

Limitations of this study include its cross-sectional nature and small number of subjects, particularly those with cognitive impairment. A longitudinal study with a larger sample will be necessary to determine if dMRI of the fornix prognosticates cognitive decline in early AD. Longitudinal analysis will also be needed to determine if imaging measures predict cognitive decline more accurately than more established biomarkers [3]. CSF and plasma biomarkers are acquired among subjects in the CADRC, but the small number of subjects who have biofluid markers and advanced imaging is even smaller than the cohort examined here. As more subjects enroll, comparison of biofluid and biomarkers will become feasible. Another limitation of this study is the large discrepancy in age between the NC group and the MCI group—a gap of nearly 10 years. While age was accounted for in the statistical analysis, it is possible that including age as a covariate masked group differences and correlations. Inclusion of age-matched subjects with NC can address this limitation.

This study is also limited by its exclusive focus on the fornix, motivated in part by the connection of fornix to hippocampus. Neurofibrillary tangles appear at the earliest stages of disease in entorhinal cortex [7], motivating investigation of age-related differences in the perforant pathway, a connection between entorhinal cortex and the hippocampus [14] by DTI [15]. The high spatial resolution required by these measurements make them challenging to perform on a routine basis. Cognitive decline might also be better conceived as a network dysfunction rather than being confined to one specific anatomical region. Whole-brain measures of functional white matter networks have been found to distinguish CN, MCI and AD groups [38]. However, the white matter pathways investigated may lack specificity to deficits in cognitive domains known to decline early in AD, such as episodic memory [5]. The use of network measures as markers of cognitive impairment remains to be seen.

There are methodological aspects that may limit the generalizability of the results. The spatial resolution and profile of diffusion weightings were chosen to adhere to standards set by ADNI3. However, these differ from those tested by Hoy et al. [31] for the DTIc model and by Zhang et al. for the NODDI model [22]. Other models, such as diffusion kurtosis imaging (DKI) were not explored, but may also prove valuable [52].

## 5. Conclusions

We have found that dMRI measures of tissue integrity in the fornix, corrected for PVA with CSF, show promise as a biomarker for cognition in AD. The next step will be to perform a longitudinal study to determine the sensitivity of the fornix measures to prognosticate cognitive decline.

## Figures and Tables

**Fig. 1.**
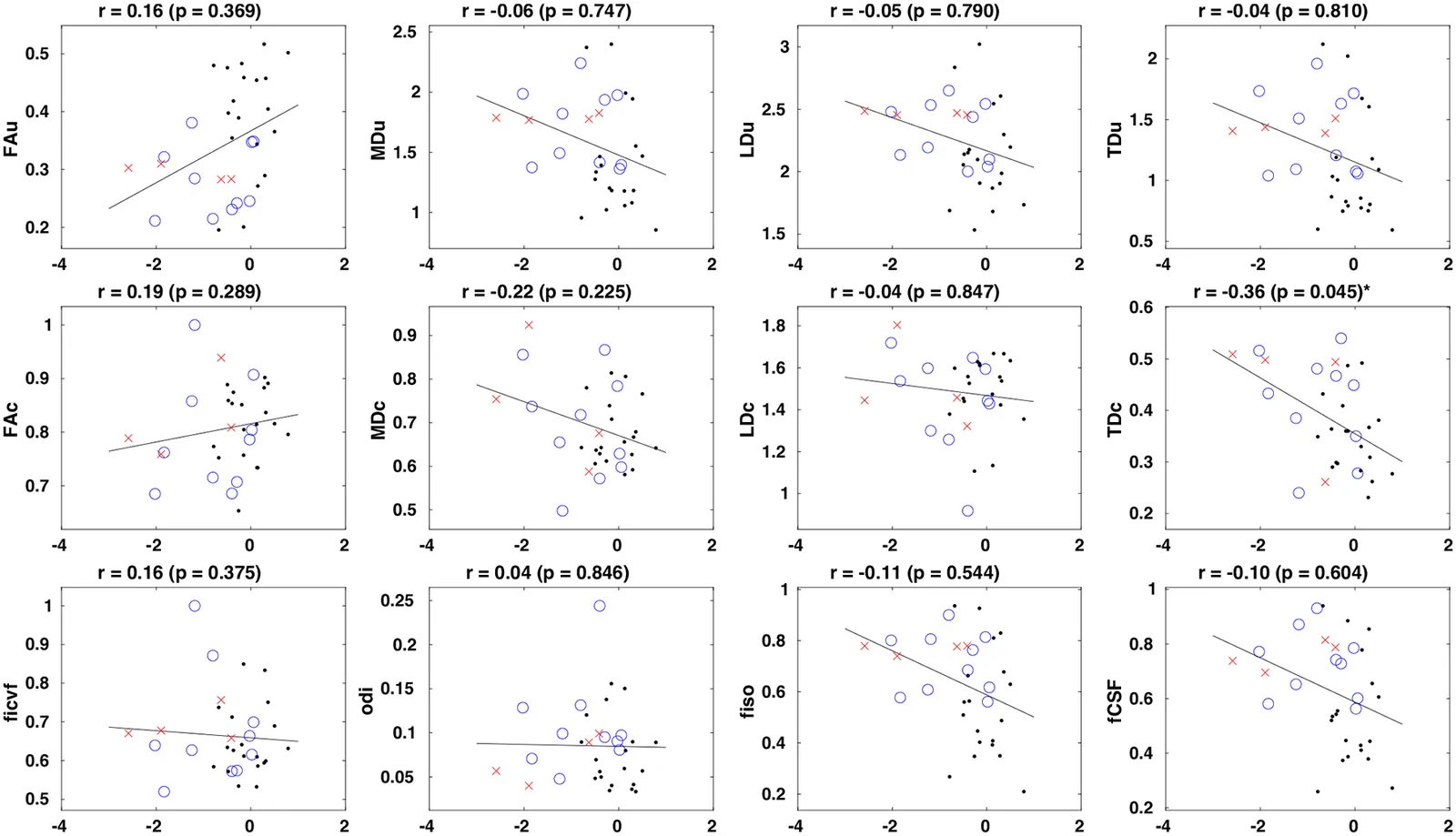
Comparison between the Universal Data Set Global Composite-5 (UDSGC5) score of cognition (horizontal axes) against dMRI values in fornix. Black dots (●), blue circles (○) and red x’s (✖) represent subjects with normal cognition (NC), mild cognitive impairment (MCI) and Alzheimer’s Disease (AD), respectively. Regression lines indicate a least squares fit to all data. The top row shows correlations with tissue integrity values (fractional anisotropy (FA), mean diffusivity (MD), longitudinal diffusivity (LD) and transverse diffusivity (TD)) from the DTIu model (uncorrected for PVA). The middle row shows correlations between tissue integrity values from DTIc (corrected for PVA). The bottom row shows correlations with NODDI parameters for tissue integrity (ficvf, odi) and fraction of signal attributed to CSF, which has isotropic diffusivity (fiso). Also in the bottom row is the fraction of signal attributed to CSF from the DTIc model (fCSF). Partial correlation coefficients are shown in the subfigure titles. Statistically significant correlations (*p* < 0.05, no correction for multiple comparisons) are marked with an asterisk (*). Units for MD, LD and TD are x10^–3^ mm^2^/s. All other tissue integrity values, as well as UDSGC5 scores, are dimensionless.

**Table 1:** Demographic characteristics of the study population. All measures show mean (standard deviation) across subjects. Subjects had Normal Cognition (NC), Mild Cognitive Impairment (MCI) or Alzheimer’s Disease (AD).

	NC	MCI	AD	p
N (female)	19 (11)	10 (3)	4 (0)	0.066
Age, years	64.4 (9.0)	73.2 (9.8)	77.5 (5.7)	0.010
Education, years	15.7 (2.7)	16.0 (2.8)	18.8 (1.5)	0.122
Neuropsychological performance				
Memory	−0.23 (0.53)	−2.30 (1.03)	−2.96 (1.07)	< 0.001
Language	−0.12 (0.71)	−1.13 (0.79)	−3.55 (2.00)	< 0.001
Attention	0.08 (0.68)	0.30 (0.60)	0.52 (0.24)	0.377
Executive Function	−0.05 (0.32)	0.06 (1.28)	0.18 (1.45)	0.875
Visuospatial	0.06 (1.13)	−0.80 (2.09)	−1.09 (1.51)	0.218
UDSGlobal5	−0.05 (0.43)	−0.77 (0.77)	−1.38 (1.04)	< 0.001
DTIu				
FA	0.39 (0.10)	0.28 (0.06)	0.29 (0.01)	0.120
MD	1.42 (0.45)	1.70 (0.33)	1.79 (0.03)	0.967
LD	2.13 (0.40)	2.31 (0.24)	2.47 (0.02)	0.901
TD	1.08 (0.45)	1.40 (0.35)	1.44 (0.05)	0.829
DTIc				
FA	0.69 (0.07)	0.64 (0.08)	0.62 (0.06)	0.428
MD	0.77 (0.10)	0.94 (0.22)	1.03 (0.19)	0.209
LD	1.53 (0.17)	1.83 (0.42)	1.96 (0.44)	0.336
TD	0.42 (0.09)	0.52 (0.18)	0.60 (0.08)	0.330
fCSF	0.51 (0.13)	0.62 (0.09)	0.65 (0.04)	0.560
NODDI				
ficvf	0.58 (0.04)	0.58 (0.06)	0.55 (0.06)	0.646
odi	0.13 (0.03)	0.15 (0.04)	0.15 (0.02)	0.515
fiso	0.52 (0.14)	0.65 (0.10)	0.67 (0.02)	0.557

**Table 2:** Comparison of cognitive domain composite scores against MDc and ficvf in fornix.

Cognitive Domain	MDc	ficvf
Memory	−0.53* (0.002)	0.35 (0.051)
Language	−0.47* (0.007)	0.29 (0.105)
Attention	0.16 (0.388)	0.10 (0.586)
Executive Function	−0.06 (0.753)	−0.04 (0.815)
Visuospatial	−0.02 (0.914)	0.33 (0.069)

Partial correlation coefficients (*p*-values) are shown. Statistically significant correlations (p < 0.05, no correction for multiple comparisons) are indicated with an asterisk (*).

## Abbreviations:

Aβ: amyloid beta
AD: alzheimer’s disease
ATN: amyloid-tau-neurodegeneration
CADRC: Cleveland alzheimer’s disease research center
CSF: cerebrospinal fluid
DKI: diffusion kurtosis imaging
dMRI: diffusion MRI
DLB: dementia with lewy bodies
DTI: diffusion tensor imaging
DTIc: diffusion tensor imaging corrected for partial volume averaging
DTIu: diffusion tensor imaging uncorrected for partial volume averaging
FA: fractional anisotropy
FAc: fractional anisotropy corrected for partial volume averaging
FAu: fractional anisotropy uncorrected for partial volume averaging
fCSF: partial volume fraction of cerebrospinal fluid
ficvf: intracellular volume fraction
fiso: isotropic diffusion volume fraction
LATE: limbic age-associated TDP-43 encephalopathy
LD: longitudinal diffusivity
LDc: longitudinal diffusivity corrected for partial volume averaging
LDu: longitudinal diffusivity uncorrected for partial volume averaging
MCI: mild cognitive impairment
MD: mean diffusivity
MDc: mean diffusivity corrected for partial volume averaging
MDu: mean diffusivity uncorrected for partial volume averaging
NC: normal cognition
NODDI: neurite orientation dispersion and density imaging
odi: orientation dispersion index
PVA: partial volume averaging
TD: transverse diffusivity
TDc: transverse diffusivity corrected for partial volume averaging
TDu: transverse diffusivity uncorrected for partial volume averaging
UDS: uniform data set
UDSGC5: uniform data set global composite 5 score
